# The Accuracy of Algorithms Used by Artificial Intelligence in Cephalometric Points Detection: A Systematic Review

**DOI:** 10.3390/bioengineering11121286

**Published:** 2024-12-18

**Authors:** Júlia Ribas-Sabartés, Meritxell Sánchez-Molins, Nuno Gustavo d’Oliveira

**Affiliations:** Departamento de Odontoestomatología, Facultad de Medicina y Ciencias de la Salud, Universidad de Barcelona, Campus Bellvitge, 08097 L’Hospitalet de Llobregat, Barcelona, Spain; julia.ribas.sabartes@gmail.com (J.R.-S.); meritxellsanchez@ub.edu (M.S.-M.)

**Keywords:** artificial intelligence, cephalometry, anatomic landmarks, cephalometry landmarks, orthodontics

## Abstract

The use of artificial intelligence in orthodontics is emerging as a tool for localizing cephalometric points in two-dimensional X-rays. AI systems are being evaluated for their accuracy and efficiency compared to conventional methods performed by professionals. The main objective of this study is to identify the artificial intelligence algorithms that yield the best results for cephalometric landmark localization, along with their learning system. A literature search was conducted across PubMed-MEDLINE, Cochrane, Scopus, IEEE Xplore, and Web of Science. Observational and experimental studies from 2013 to 2023 assessing the detection of at least 13 cephalometric landmarks in two-dimensional radiographs were included. Studies requiring advanced computer engineering knowledge or involving patients with anomalies, syndromes, or orthodontic appliances, were excluded. Risk of bias was assessed using Quality Assessment of Diagnostic Accuracy Studies (QUADAS-2) and Newcastle–Ottawa Scale (NOS) tools. Of 385 references, 13 studies met the inclusion criteria (1 diagnostic accuracy study and 12 retrospective cohorts). Six were high-risk, and seven were low-risk. Convolutional neural networks (CNN)-based AI algorithms showed point localization accuracy ranging from 64.3 to 97.3%, with a mean error of 1.04 mm ± 0.89 to 3.40 mm ± 1.57, within the clinical range of 2 mm. YOLOv3 demonstrated improvements over its earlier version. CNN have proven to be the most effective AI system for detecting cephalometric points in radiographic images. Although CNN-based algorithms generate results very quickly and reproducibly, they still do not achieve the accuracy of orthodontists.

## 1. Introduction

Artificial Intelligence (AI) is defined as the ability of a computer to perform tasks that are typically undertaken by humans, attempting to mimic their logic, intelligence, and discernment [1].

To better understand how this technology operates, it is essential to introduce the learning methods currently employed and their modes of action, enabling AI to perform the tasks it can accomplish today.

- Machine learning (ML) is a subset of AI and can be considered the central axis. Its functioning is based on the exposure to multiple sample data and their desired outcomes. This aims to “train” the algorithm, and through a set of probability and statistical tools, allows the machine to automatically detect new patterns and solve problems on its own [1,2,3].
- Deep learning (DL) is a part of machine learning. It seeks to mimic the human cognitive system by creating an artificial neural network with multiple layers, aiming to create a system that analyzes data and identifies more complex patterns than initially presented to improve its detection [2,4]. The learning method involves exposing the algorithm to pairs of data and their corresponding labels, such as relating a cephalometric point to its definition repetitively, so that it can predict the labeling of new data in the future [1]. While deep learning includes other types of models, such as recurrent neural networks (RNNs) for sequential data or transformers for language, CNNs are specifically designed to recognize spatial hierarchies in data, making them well-suited for tasks like image classification, object detection, and segmentation. Today, the most commonly used method for detection in radiological tests is CNN [1].

As early as 1950, scientist Alan Turing, one of the founders of modern computers, first described artificial intelligence. His goal was to create computers that imitated human behavior, highlighting their intelligence and critical thinking. In his famous “Turing Test”, he attempted to converse with a computer to determine if it could think and reason [5]. Six years later, in 1956, John McCarthy, a computer scientist and cognitive scientist, defined AI at the Dartmouth College [1] conference as the science and engineering of making intelligent machines [6]. From then on, the path toward this direction began to open in many areas of life, including medicine. In Dentistry, AI has also played a significant role, contributing to radiographic interpretation, as well as the detection of dental caries, cysts and tumors, histological studies, evaluating growth and development, and predicting treatment outcomes [7]. It is important to note that the role of the professional remains entirely necessary. Orthodontics requires very meticulous precision, as the results usually generate irreversible changes [8], so it is the professional who must continue to provide their own knowledge acquired from extensive training and experience to achieve a final diagnosis and corresponding treatment plan [2].

Among the most promising applications of AI in orthodontics are the decision to perform dental extractions, the classification of malocclusions using three-dimensional imaging systems such as cone beam computed tomography (CBCT), the evaluation of skeletal age, the prediction of growth patterns, and the planning of orthognathic surgeries. Technology has also facilitated the precise location of cephalometric points, among other functions [1,2].

Tracing it back to its beginning, cephalometry was introduced by Broadbent and Hofrath in 1931 [9] in the field of orthodontics. Its purpose was to stop making diagnoses based solely on clinical observation and dental casts and begin analyzing malocclusions from a much deeper perspective, relating them to skeletal, facial, and dental morphology. Since then, cephalometric study has become the standard diagnostic method for clinical practice and research in orthodontics.

To perform a cephalometric analysis, it is necessary to start by making a very detailed detection of the anatomical points in the craniofacial region from a radiological image. Traditionally, this is a two-dimensional lateral cephalometric radiograph of the skull, although three-dimensional images are increasingly being used [10]. This process requires a considerable investment of time and is usually challenging for two main reasons: first, it is laborious to obtain good projections of the craniofacial region in two dimensions due to the superposition of structures; secondly, there is significant anatomical diversity among patients [11].

The importance of correctly positioning these anatomical references is paramount as they directly influence the case analysis and treatment decision. An incorrect location by just a few millimeters or degrees can cause a wrong classification of the pathology, leading to poor practice as a result [10].

To try to avoid the variability in positioning between observers due to the lack of certainty in the exact location of the points, and the intra-observer error that can be generated by the fatigue caused by manual execution and the time it entails, it is necessary to reconsider the current situation and provide solutions to professionals.

In this context, the emergence of semi-automated programs [11,12] has marked a significant advance, but it has been recently recognized that there is a need for a fully automated tool based on AI that can perform these tasks with consistent precision and high reliability, processing large volumes of data efficiently and eliminating the inconsistencies inherent in human intervention [10].

Over the last few years, a large body of literature has been published on the detection of cephalometric points, which has made it evident that there is a need to filter and classify the information. Furthermore, although AI is a tool that promises great functionality, it still has certain limitations, such as difficulty in achieving precision in detection when there is variability in the image, differences in the demographics of the subjects, heterogeneity in the characteristics of malocclusion [13], overfitting in the data used for training, and erroneous results in the test data [14], among others. Given these circumstances, the motivation for carrying out this systematic review lies in deepening and establishing the existing knowledge, as well as in addressing the present unknowns.

Current evidence suggests that the most widely used and best-performing form of artificial intelligence for detecting cephalometric points in two-dimensional images is CNN using the deep learning method. Additionally, the algorithm currently associated with the lowest error rate is believed to be You Only Look Once Version 3 (YOLOv3), which also utilizes deep learning. However, while manual localization of points remains more accurate than automated methods, artificial intelligence demonstrates better reproducibility compared to detection performed by professionals.

The main objective of this study is to identify the artificial intelligence algorithms that yield the best results for cephalometric landmark localization, along with their learning system. Secondary objectives include analyzing the most commonly used AI algorithms today, comparing the accuracy of cephalometric point detection by AI with that by professionals, and determining the reproducibility of AI compared to manual tracing.

## 2. Materials and Methods

The protocol for this review was conducted following the Preferred Reporting Items for Systematic Reviews and Meta-Analyses (PRISMA) [15] protocols and was registered with PROSPERO under the registration number CRD42024599610.

### 2.1. Focused Question

What forms of AI have the lowest error and highest reproducibility in detecting cephalometric landmarks in lateral cranial teleradiography compared to professionals?

### 2.2. Eligibility Criteria

A PICO question (Table 1) was designed to determine the eligibility criteria and to enable us to answer the research question.

The types of studies included were diagnostic accuracy studies (DASs) and cohort observational studies published in the last 10 years (2013–2023) that exclusively evaluated the detection of cephalometric landmarks using artificial intelligence for orthodontics in lateral cephalometric radiographs, provided these were not derived from 3D images such as CBCT. All studies analyzing at least 13 cephalometric landmarks in soft and/or hard tissue and performing any type of analysis on AI methods or comparisons of these were considered. Similarly, comparisons between AI methods and/or conventional analysis methods were accepted. Studies providing results such as mean error, accuracy, sensitivity, and reproducibility were also included.

Case–control studies, case series, cross-sectional studies, case reports, personal opinions, letters to the editor, and systematic reviews were not considered. Studies including patients with craniofacial anomalies or syndromes or patients undergoing orthodontic treatment were excluded. Articles focusing on complete cephalometric analysis rather than the exclusive detection of landmarks, those too specific to AI making them difficult for the authors to interpret, and those with purposes other than orthodontics were also excluded. Finally, articles not available in full text were excluded.

No restrictions were applied regarding patient age or language.

### 2.3. Search Strategy and Information Sources

An electronic literature search was performed in PubMed-MEDLINE, Cochrane, Scopus, IEEE Xplore, and Web of Science between November and December 2023, and the search strategy applied across the different electronic databases was based on a combination of Medical Subject Headings (MeSH) and free-text terms found in titles and abstracts. The keywords used were “artificial intelligence”, “cephalometry”, “anatomic landmarks”, “cephalometry landmarks”, and “orthodontics”, combined with the Boolean operators AND and OR. The strategy employed for each data source is shown in Table 2.

### 2.4. Study Selection and Data Extraction

The selection of studies was conducted by one researcher (R-S.J) in three phases, following the selection steps outlined in the PRISMA [15] statement, and was subsequently reviewed by a second researcher (d’O.NG).

First, duplicate articles were removed. Next, the remaining articles were screened by title and abstract, excluding only those references that provided information irrelevant to the research question. In the second phase, articles that did not provide sufficient information for a decision to exclude were retrieved in full text to assess their eligibility based on the pre-established criteria. Finally, the full-text articles were read and selected according to the same criteria used in the second phase.

The data extraction process was conducted by R-S.J and checked by d’O.NG Any disagreements were resolved through careful discussion.

The information gathered from each reference was classified according to: study identification (author, year, country of publication), study design, originating institution, objectives, number of participants, age and gender, algorithm and learning method, number and location of cephalometric landmarks, number of images for training, validation, and testing, gold standard, success detection rate (SDR) within a clinical range of 2 mm expressed as a percentage, mean radial error (MRE) and standard deviation (SD) expressed in millimeters, and finally, other possible outcomes.

### 2.5. Risk of Bias

A quality assessment was carried out by two reviewers, R-S.J and d’O.NG to determine the risk of bias of the included studies.

The tool used for the diagnostic accuracy study was QUADAS-2 to assess the risk of bias and concerns regarding applicability [16]. The following domains were analyzed: (1) patient selection; (2) index test (s); (3) reference standard; (4) flow and timing.

Publications were categorized as follows: (A) low risk of bias (bias that does not seriously affect the results); (B) high risk of bias (bias that undermines the reliability of the results); (C) unclear risk of bias when there were very few details available to classify as “high” or “low” risk.

For comparative cohort observational studies, NOS [17] was implemented, which evaluates studies based on three categories: (1) Selection, (2) Comparability, and (3) Outcome. Each category was scored according to a number of specific evaluations, with a maximum total of 9 stars. A study with a score of 7 or more stars was considered to have a low risk of bias, while a score below 7 stars indicated a high risk of bias.

## 3. Results

### 3.1. Study Selection

**Figure 1:**
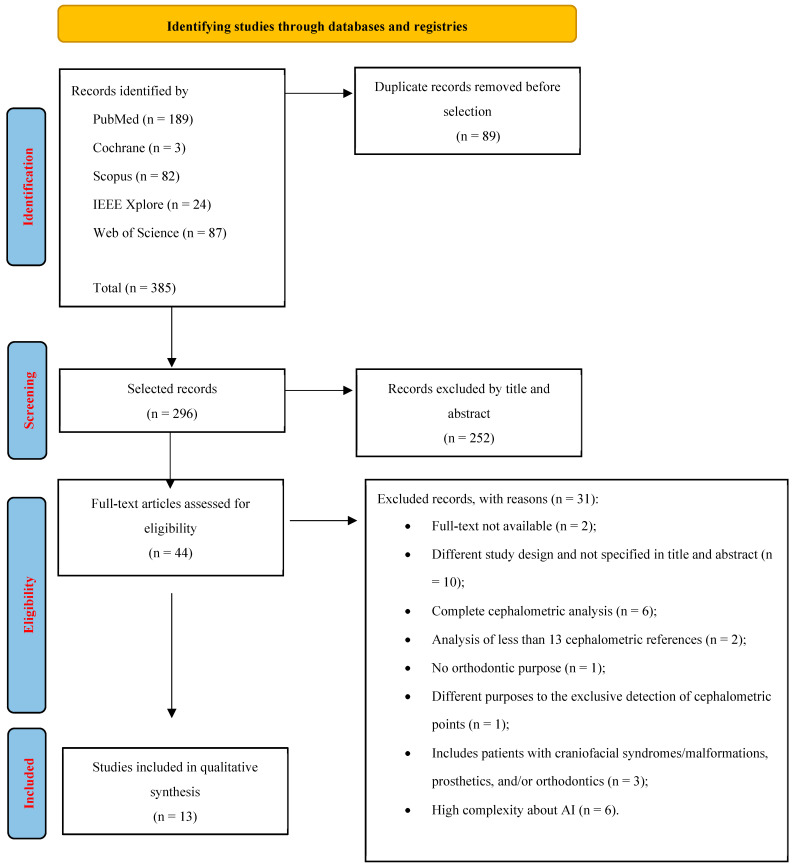
PRISMA flowchart of search strategy and study selection.

The flowchart of the study selection process is shown in Figure 1. The initial electronic search revealed a total of 385 records, with 89 references being duplicates. After reviewing the titles and abstracts, 44 studies were selected for a more detailed examination, resulting in the exclusion of 252 studies. Subsequently, 31 articles were discarded for not meeting the selection criteria [9,18,19,20,21,22,23,24,25,26,27,28,29,30,31,32,33,34,35,36,37,38,39,40,41,42,43,44,45,46,47], and no additional studies were found through manual search. Finally, the application of the inclusion criteria allowed for a total of 13 references to be included in the qualitative review.

Throughout the process, there was consensus between the authors regarding the selection and classification of the literature.

### 3.2. Risk of Bias Assessment for Diagnostic Accuracy Studies

**Figure 2:**
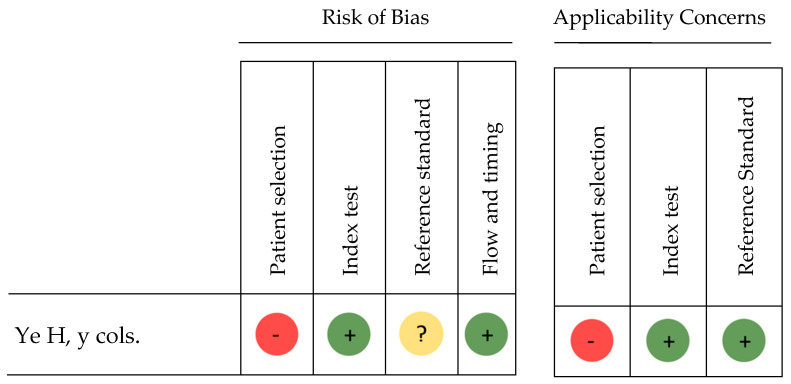
Risk of bias for diagnostic accuracy studies [48].

Figure 2 shows the risk of bias assessment for the included reference classified as a diagnostic accuracy study, performed using the QUADAS-2 tool [16]. As shown in the figure, the symbol (+) indicates low risk of bias, the symbol (?) indicates unclear risk of bias, and (–) represents high risk.

Domain 1: Patient Selection

In terms of risk of bias, this study [48] was considered to have a high risk in patient selection. Although the selection was conducted randomly and the patients were representative of the context in which the test is intended to be applied, some were excluded due to pathological conditions such as cleft palate or craniofacial syndromes. Accordingly, the applicability of this study was also rated as high risk.

Domain 2: Index Test

The index test was performed consistently and applied to all patients without variability, resulting in a low risk of bias. However, the applicability risk was rated as unclear due to insufficient details about the algorithms used for the AI systems.

Domain 3: Reference Standard

The reference standard was applied by consensus among three experts; however, inter-rater consistency was not assessed, leading to an unclear risk of bias for this domain. The applicability of the reference standard was considered low risk.

Domain 4: Flow and Timing

Both the index test and reference standard were applied to all patients without any losses, and the timing was appropriate. This domain was rated as having a low risk of bias for both applicability and overall bias.

### 3.3. Risk of Bias Assessment for Cohort Observational Studies

Table 3 shows the risk of bias assessment for cohort observational studies according to NOS [17]. According to the table, a total score of less than seven stars indicates a high risk of bias, while a total score of seven stars or more is considered to indicate a low risk of bias. Based on this assessment scale, seven studies [10,49,50,51,52,53,54] were classified as low risk, and five studies [55,56,57,58,59] were classified as high risk.

### 3.4. Data Extraction: Qualitative Synthesis

The information extracted from the selected publications for this review is summarized in Table A1 and Table A2, which are included in the Appendix A. These tables analyze, whenever possible, the study design and institutional origin, objectives, and participant characteristics; the AI algorithm and its learning method; the number and location of cephalometric landmarks; the characteristics of the images used as well as the comparison method with the gold standard; and finally, the results in terms of success rate (SDR), mean error (MRE), or other relevant outcomes.

Among the 13 selected articles, seven authors [10,49,51,55,56,58,59] aimed to develop and test new algorithms to be compared with the manual localization of cephalometric landmarks performed by professionals. Results were reported in terms of mean successful detection within a clinical range of 2 mm and mean error, with values ranging from 64.3% to 97.30% and from 1.04 mm ± 0.89 to 3.40 mm ± 1.57, respectively. Notably, the SDR value reported by Kim YH et al. [58] was the lowest, testing a fully automatic model based on CNN on 950 cephalograms and 13 hard tissue points. In contrast, Kim J et al. [51] and Ramadan R et al. [49] also employed models for detecting regions of interest (ROI) and achieved higher percentages, ranging from 83.6% to 90.39%. Yao J et al. [55] achieved the highest success rate with an algorithm based on a global detection module and a locally modified module, tested on 512 radiographs and 37 cephalometric landmarks in both hard and soft tissue. Regarding mean error, Yao J et al. [55] also obtained the best value, while Uğurlu M [56], testing the CranioCatch algorithm on 1620 cephalograms and 21 soft and hard tissue points, achieved the highest error.

On the other hand, with a different objective, five studies [48,50,52,53,57] aimed to test algorithms and compare their results with the same gold standard as the previous ones, namely, manual tracing. Consequently, the results differed from those previously mentioned. Ristau B et al. [50], Bulatova G et al. [52], Santos Menezes L et al. [53], and Davidovitch M et al. [57] focused on measuring the differences in landmark localization between AI and conventional methods according to the *x*/*y* coordinate axes. Santos Menezes L et al. [53] also modified brightness and contrast conditions. For the x-axis, the best-located points were Nasion [52] and Gonion [53], while the worst were Porion [50,53], Gonion [52], Orbital, Ptm, and Basale [57]. For the y-axis, the least differing references were Nasion [52], Pogonion [53], nose tip, and point B [57], while the worst localized were the apices of lower incisors [50,52], Subnasal [53], Orbital [50], Porion [50,57], soft Pogonion, and upper lip [57].

Ye H et al. [48] evaluated three AI methods (MyOrthoX, Angelalign, and Digident) on a sample of 33 cephalograms and 32 landmarks from both tissue types in terms of SDR and MRE for each algorithm in clinical ranges of 1 and 2 mm, achieving promising results with Angelalign, with a successful detection rate of 93.09% and a mean error of 0.80 mm ± 0.26.

Finally, Zhao C et al. [54] evaluated the new Multi Scale-YOLOV3 algorithm, comparing it quantitatively and qualitatively with YOLOv3 as the reference standard. In this study, using a sample of 400 cephalograms and 19 landmarks from both soft and hard tissue, the success rate improved by 3.5% for the new model. Although mean error was not reported, it was noted that both minimum and maximum errors for cephalometric landmark localization were better for MS-YOLOv3.

Additionally, it is noteworthy that all the collected studies employed AI systems using the same learning method, namely, DL and CNN, except for Lee J et al. [10], whose method was based on Bayesian convolutional neural networks (B-CNN).

## 4. Discussion

In this study, the aim was to analyze the most prominent artificial intelligence (AI) methods currently available for automating the detection of cephalometric landmarks. Our goal was to understand their limitations and advantages in order to provide orthodontists with tools that simplify their work and effectively address current patient demand.

Recent studies have validated the existence of systems capable of producing tracings very similar to those performed by professionals. A notable example is the work by Yao J et al. [55], who, in 2022, developed an algorithm based on a global detection module and a locally modified module, achieving a level of precision close to the gold standard, with results of 97.30% and 1.04 mm ± 0.89 in terms of SDR and MRE, respectively. These values could be explained, among other factors, by the exclusion criteria applied in the study, which avoided cephalograms from patients with conditions that could hinder accurate detection, such as cleft lip or palate, orthodontic appliances, among others. However, CNN-based models tested in the research by Kim J et al. [51], Lee J et al. [10], Ramadan R et al. [49], and Uğurlu M [56] also achieved promising SDR values ranging from 76.2% to 90.39%, and MRE values ranging from 3.4 to 1.23 mm.

B-CNN represent a promising option for improving the localization of cephalometric landmarks. Unlike more common approaches such as CNNs, this option does not limit itself to learning solely from training data but also considers the uncertainty associated with its predictions. This factor is particularly relevant in fields where precision and confidence in results must be excellent, as demonstrated by Lee J et al. [10] on the public dataset established at the 2015 IEEE International Symposium on Biomedical Imaging.

On the other hand, models based on the concept of creating regions of interest (ROI) also demonstrated very good precision [49,51,58]. This process involves identifying areas where cephalometric landmarks would be located based on distinctive features and then determining their coordinates. Although Kim YH et al. [58] did not achieve clinically valid results, Kim J et al. [51] and Ramadan R et al. [49] did, with an average success rate of up to 86.5%. However, evidence from Hwang JJ et al. [60] highlights a limitation of this approach, as it prevents the system from learning from the entire image and instead focuses on a small portion. Similarly, Kim J et al. [51] emphasized the importance of generalization when selecting training images to avoid overfitting and, consequently, unrealistic results. This issue can arise when the dataset is limited; therefore, the study utilized a total of 3150 radiographs from 10 hospitals with different types of radiographic machines, image qualities, and patient characteristics, which contributed to the validity of their findings.

The YOLOv3 algorithm was tested by Bulatova G et al. [52] with 110 radiographs for hard tissue points, and also by Hwang H et al. [59], who trained it with 1983 cephalograms and tested it with 200, to detect both hard and soft tissue points. The results were almost identical in both cases, with 75% of the points correctly identified. In fact, Hwang H et al. [19], in 2020, also demonstrated with this same algorithm and an automatic framework of 80 cephalometric points, a precision and reproducibility very similar to human performance, concluding that this solution is a viable option for identification.

Recently, Zhao C et al. [54] conducted a comparison between YOLOv3 and an improved version called MultiScale-YOLOv3. It is noteworthy that the findings showed a slight improvement for this new variant. For both versions, the point with the lowest success rate was Gonion, typically due to overlapping areas. However, for MS-YOLOv3, the point with the lowest error was Pogonion soft tissue, contrasting with the higher error rate observed for YOLOv3 at the same point. This finding could be considered an improvement for this new variant, as soft tissue points are usually challenging to identify due to their lack of contrast. In this case, there is an improved error trend for this cephalometric reference with MS-YOLOv3.

When testing AI for analysis according to *x*/*y* coordinate axes [50,57], different studies reached varying conclusions, with no clear trend of results observed among them. Ye H et al. [48] compared the tracing accuracy of automatic programs and found that the results of MyOrthoX, Angelalign, and Digident were very close to clinically acceptable standards, with success rates between 93% and 89.99% and errors ranging from 0.8 to 1.11 mm. This study also considered the time required and observed no significant difference between the algorithms, nor an unequivocal trend favoring one modality over another in terms of time savings. However, the time was substantially shorter compared to professionals, which aligns with findings from other researchers.

Among the conditions that could influence accurate landmark localization, Kim J et al. [51] referred to image quality. A significant correlation was observed between examiners and the deep learning model’s accuracy in locating the landmarks, suggesting that cephalogram image quality affects precision for both. Image quality could depend on factors such as focus-to-receptor distance, sensor type, manufacturer, etc. Specifically, it was observed that higher tube tension, longer exposure time, and smaller sensor size led to decreased performance [51]. Meanwhile, Santos Menezes L et al. [53] analyzed the impact of brightness and contrast adjustments on CEFBOT detection and concluded that marking errors were frequent when contrast was high and brightness was low, especially in the identification of soft tissues.

Locating cephalometric landmarks in hard and/or soft tissue is a factor that affects their accurate detection. A consistent pattern was observed across the 13 studies reviewed, with landmarks located in hard tissue demonstrating a higher success rate than those in soft tissue. Soft tissues generally have poorer boundaries and lack distinctive features compared to hard tissues [10,48,49,54,55]. Ye H et al. [48] also attributed this to areas with higher darkness or lower brightness. The landmark with the most significant overall error was Pogonion soft tissue.

Regarding hard tissue landmarks, those located in bilateral or overlapping cranial structures were particularly challenging to identify [50,51,55]. According to evidence gathered by Durão AP et al. [61], landmarks such as Porion, Condylion, Orbital, Basion, Gonion, Anterior Nasal Spine, and Posterior Nasal Spine are particularly prone to errors. During this review, it was noted that the Pterygoid landmark might overlap with the pterygomaxillary fissure, while Gonion could be confused with the mandibular ramus [54,55]. On the other hand, Articulare would be challenging due to its location at the junction of the mandibular condyle and the external dorsal contour of the temporal bone [10,54], and Porion could be confused with the auricular structure of the internal auditory canal [48,50,54]. Additionally, anatomical references in curved areas, such as point A [10] situated in the premaxilla or point B in the chin [51], also faced difficulty due to a lack of distinctive features, such as intersections with other lines [10,51,55,58]. However, according to Yao J et al. [55], the error associated with the Chin point was attributed more to the lack of chin development than to the curvature of the area. Dental landmarks, whether in the apical or coronal region, can present identification challenges due to the presence of open apices, dental crowding, or lack of contrast with surrounding bone [55].

Finally, when evaluating the x and y axes, no definitive conclusions were reached regarding which axis exhibited lower precision.

In this analysis, the researchers established a clinical threshold of 2 mm to assess the precision of artificial intelligence, as this was the most commonly used range in the reviewed literature. However, the authors of this study question the suitability of this criterion, arguing that it might encompass too broad a margin of error to be considered acceptable. According to scientific evidence [61,62], a range of 1 to 2 mm could be deemed valid without compromising diagnosis and, consequently, treatment planning.

In three of the selected articles [48,51,55], the performance of algorithms was examined within ranges of 1 mm or less, showing a significant decrease in the average success rate, ranging from 54% to 60%. These figures are considered too low for clinical application. The Angelalign algorithm, used by Ye H et al. [48], stood out with a success rate of 78%, even surpassing other AI models at greater distances. These findings highlight the need to continue refining artificial intelligence systems to achieve precision comparable to that of orthodontists.

### 4.1. Limitations

Our review may have certain limitations, starting with two of the exclusion criteria applied. Studies focusing on highly specific or advanced levels of expertise in artificial intelligence were excluded, which may have led to missing valuable data. This criterion was motivated by the aim to ensure that the authors could fully understand the subject matter and provide a more general and accessible approach for our target audience: orthodontic professionals. Additionally, only articles published in the last 10 years were considered to ensure that this study reflects the most recent and updated literature. However, this temporal restriction might have led to the omission of classic or fundamental works on the topic.

### 4.2. Future Investigations

Future research should aim to continue exploring the integration of artificial intelligence by including a broader range of specialized studies and utilizing larger datasets with greater heterogeneity in conditions, such as overlapping and varying quality images, differences in skeletal structures, or different dental statuses, thereby achieving more generalized results.

## 5. Conclusions

- Our findings suggest that CNNs represent the most promising AI form for detecting cephalometric landmarks in 2D lateral cranial teleradiography, offering lower error rates and higher reproducibility compared to other AI types reviewed.
- However, due to significant heterogeneity in study designs, data collection, and performance metrics, a definitive quantitative comparison was not feasible
- While AI demonstrates faster and more reproducible results than manual tracing, no algorithms currently match the precision of human professionals.
- Future research should aim to standardize evaluation criteria and datasets to enable a more robust comparison of AI methods.

## Figures and Tables

**Table 1:** PICO (P, population; I, intervention; C, comparison; O, outcomes).

PICO Question for Inclusion Criteria	
P (Population)	Orthodontic patients with lateral cranial radiographs (2D)
I (Intervention)	Automatic detection of cephalometric landmarks by artificial intelligence (AI) models
C (Comparison)	Reference standards established by professionals and the existing literature
O (Outcomes)	Measurable or predictive outcomes, such as accuracy, sensitivity, and reproducibility

**Table 2:** Search strategy by databases.

Databases	Search Strategy
Pubmed	((artificial intelligence [Title/Abstract] AND anatomic landmarks [Title/Abstract]) OR cephalometry [Title/Abstract] OR cephalometry landmarks [Title/Abstract]) AND orthodontics [Title/Abstract] AND (“2013” [Date—Publication]: “2023” [Date—Publication])
Scopus	artificial AND intelligence AND cephalometric AND landmarks
Cochrane	artificial intelligence AND orthodontic appliance
IEEE Xplore	artificial intelligence AND cephalometric landmarks
Web of Science	((TS = (artificial intelligence)) AND TS = (cephalometric landmarks)) and 2023 or 2022 or 2021 or 2020 or 2019 or 2016 (Publication Years)

**Table 3:** Risk of bias assessment according to Newcastle–Ottawa Scale for observational cohort studies.

	Selection				Comparison		Outcomes			
Articles	Representa-Tiveness of the Exposed Cohort	Unexpo-sed Cohort Selection	Exposure Determination	Demonstration of an Aspect of Interest not Present at the Start of the Study	Study Controls	Study Controls for Any Additional Factors	Evaluation of the Result	Sufficiently Long Follow-Up Time	Adequacy of Cohort Monito-ring	Conclusions
Kim Y, et al. [58]		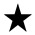	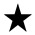		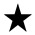		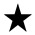	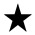	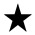	(6) HIGH
Kim J, et al. [51]		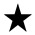	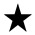	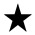	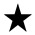		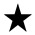	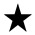	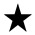	(7) LOW
Bulatova G, et al. [52]	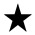	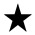	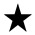	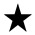	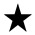		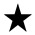	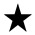	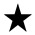	(8) LOW
Santos Menezes L, et al. [53]	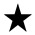	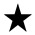	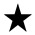		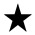	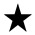	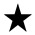	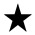	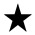	(8) LOW
Lee J, et al. [10]	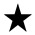	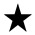	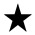		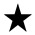		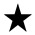	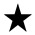	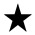	(7) LOW
Yao J, et al. [55]		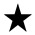	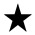		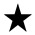		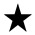	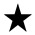	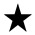	(6) HIGH
Zhao C, et al. [54]	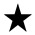	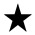	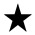		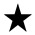		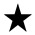	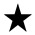	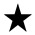	(7) LOW
Ristau B, et al. [50]	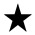	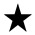	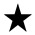		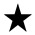		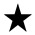	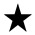	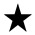	(7) LOW
Ramadan R, et al. [49]	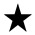	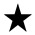	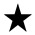		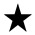		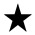	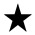	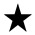	(7) LOW
Hwang H, et al. [59]		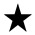	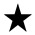		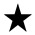		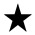	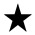	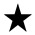	(6) HIGH
Uğurlu M [56]		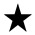	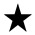		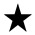		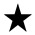	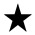	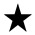	(6) HIGH
Davidovitch M, et al. [57]		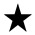	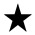		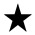		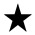	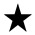	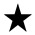	(6) HIGH

The NOS includes 3 categorical criteria with a maximum of 9 points/stars. A score of ≥ 7 stars is considered as “low risk of bias”; a score of ≤ 6 stars is considered as “high risk of bias”.

## Data Availability

The data that support the findings of this study are available from the corresponding author upon reasonable request.

## Appendix A. Data Collection Tables

**Table A1:** Summary table of the articles included in the systematic review.

Author Origin Year	Study Design	Originating Institution	Objectives	Number of Participants Age Gender Other
Kim YH, et al. [58] Korea 2021	RCC	Yonsei University Dental Hospital	Testing fully automatic point localization model based on CNN	950 RX Age: - Gender: -
Kim J, et al. [51] Korea 2021	RCC	10 university hospitals in South Korea	Investigating the accuracy of automated identification of lateral cephalometric points using RetinaNet as CNN	3.150 RX Age: - Gender: -
Bulatova G, et al. [52] USA 2021	RCC	College of Dentistry, University of Illinois	Comparing the accuracy of CNN-based YOLOv3 algorithm with manual localization performed by experts	110 RX Age: - Gender: -
Santos Menezes L, et al. [53] Brazil 2023	RCC	Federal University of Bahia, Piracicaba Dental School, University of Campinas, University of São Paulo, Federal University of Sergipe, Brazil	Evaluation of CEFBOT AI software to identify cephalometric points in four brightness and contrast conditions	30 RX Age: >18 years old Gender: - Others: without serious craniofacial deformities and asymmetries, unerupted incisors and teeth impacted on the apex of the incisors.
Lee J, et al. [10] Korea 2020	RCC	Ewha Womans University Medical Center Seoul	Testing a BCNN-based model for cephalometric landmark localization with uncertainty-based confidence regions	400 RX (ISBI public data IEEE 2015) Age: 6 to 60 years old Gender: -
Yao J, et al. [55] China 2021	RCC	West China School of Stomatology, Sichuan University	Developing a new system for the automatic localization of cephalometric points, based on CNN	512 RX Age: 9 to 40 years old Gender: 247 men, 265 women Others: No patients with absent, atypical or missing central incisors, molars, orthodontic wearers, cleft lip or palate, maxillofacial trauma or candidates for surgery or implants.
Zhao C, et al. [54] China 2023	RCC	Jiading Central Hospital, Shanghai University of Medicine and Health Sciences	Evaluation of the MS-YOLOv3 algorithm for automatic landmark detection, and quantitative and qualitative comparison with YOLOV3	400 RX (ISBI public data IEEE 2014) Age: 6 to 60 years old Gender: -
Ristau B, et al. [50] USA 2022	RCC	Louisiana State University School of Dentistry, New Orleans	Comparing the reliability of AudaxCeph for identifying cephalometric landmarks with human trackers	60 RX Age: - Gender: - Others: Complete dentition, without craniofacial abnormalities, asymmetries or significant double borders of the mandible
Ramadan R, et al. [49] Saudi Arabia 2022	RCC	Faculty of Engineering and Computer Science, Ha’il University Faculty of Computing and Artificial Intelligence, Benha University Department of Computer Engineering, Balkan International University,	Testing deep learning model based on centroid registration and ResNet50	400 RX (ISBI public data IEEE 2015) Age: 7 to 76 years old Gender: 235 women, 165 men
Hwang H, et al. [59] South Korea 2021	RCC	Seoul National University Dental Hospital	To compare and evaluate fully automated cephalometric point localization based on a new form of YOLOv3 with manual tracing	2.183 RX Age: - Gender: -
Ye H, et al. [48] China 2023	RCT	Chongqing Medical University	Compare the accuracy of automatic or AI-assisted program layout; MyOrthoX, Angelalign and Digident with manual localization	33 RX Age: - Gender: - Others: No patients with cleft lip and palate, systemic diseases or craniofacial syndromes
Uğurlu M [56] Türkiye 2022	RCC	Faculty of Dentistry, Eskişehir Osmangazi University	Develop AI model (CranioCatch) for automatic detection of cephalometric points	1.620 RX Age: 9 to 20 years old Gender: -
Davidovitch M, et al. [57] Israel 2022	RCC	University Orthodontic Clinic Tel Aviv University	Evaluation of automatic recognition of cephalometric points by the Algoceph convolutional neural network (CNN) artificial intelligence system	10 RX Age: - Gender: - Other: No congenital craniofacial/dental anomalies or facial trauma

RCC: retrospective comparative cohort; DAS: Diagnostic Accuracy Study; AI: artificial intelligence; CNN: convolutional neural networks; BCNN: Bayesian convolutional neural networks; YOLOv3: You Only Look Once version 3; MS-YOLOv3: Multi-Scale You Only Look Once version 3.

**Table A2:** Summary table of the characteristics of the articles included in the systematic review with respect to the intervention and results.

	INTERVENTION				OUTCOMES		
	Algorithm Learning Method	Cephalometric Landmarks: Number Location	Images: Training Validation Test	Gold Standard	SDR Clinical Range 2 mm	MRE +/- SD	Others
Kim YH, et al. [58]	ROI machine; detecting areas of interest with points, and detection machine to predict coordinates CNN, DL	13 points Hard tissue	800 training 100 validation 50 test	ML performed by 2 orthodontists with 15 and 5 years of experience	Mean SDR: 64.3% Cephalometric point with the **highest** success rate: UIB y LIB 78.7% Cephalometric point with the **lowest** success rate: Ar 46.7%	Mean MRE: - Cephalometric point with the **highest** mean error: S 2.39 mm Cephalometric point with the **lowest** mean error: UIB 1.36 mm	-
Kim J, et al. [51]	Fully automated prediction algorithm based on 2 phases: Region of Interest (ROI) detection and landmark prediction CNN, DL	20 points Hard tissue	1.392 training 1218 additional training and development 440 validation 100 test	ML performed by 2 orthodontists with 30 and 10 years of experience	Mean SDR: 83.6% Cephalometric point with the **highest** success rate: S 100% Cephalometric point with the **lowest** success rate: Mx6 root 65%	Mean MRE: 1.36 mm +/- 0.98 Cephalometric point with the **highest** mean error: Md6 distal 2.09 mm +/- 1.91 Cephalometric point with the **lowest** mean error: Crown Mx1 0.46 mm +/- 0.37	SDR clinical range 0–1 mm Mean SDR: 56.55% Cephalometric point with the **highest** success rate: S 98% Cephalometric point with the **lowest** success rate: B 26%
Bulatova G, et al. [52]	YOLO version 3 CNN, DL	16 points Hard tissue	- No algorithm development	ML performed by 2 orthodontists	-	-	Absolute difference between AI and MT according to X, Y coordinate axes ***p* > 0.05 (no significative diference):** 75% points ***p* < 0.05 (significative diference):** 25% points **Eje X:** Point that differs the most: Go absolute value 8.7 +/- 9 Point that differs the least: Na absolute value 1.4 +/- 3.9 **Eje Y:** Point of greatest difference: L1apex absolute value 5.4 +/- 2.9 Point that differs the least: Na absolute value 1.3 +/- 2.7
Santos Menezes L, et al. [53]	CEFBOT CNN, DL	19 points 15 Hard tissue 4 Soft tissue	- No algorithm development	ML performed by 2 examiners, ECont (control) and ECal (calibration)	-	-	Reliability. Mean ICC between AI and MT for four brightness and contrast settings: **X axis**: ICC > 0.93 (very good). Best outcome AI with V3, worst with V4. **Y axis:** ICC > 0.89 (good). Best outcome AI with V3, worst with V4. Reproducibility. *p* < 0.05 between AI and MT for four brightness and contrast settings: **X axis:** Point with **highest error:** Po (*p* < 0.001) in V1, V2, V3, V4, V5. Point with **lowest error:** V1 Pog (*p*: 0.949); V2 Ll (*p*: 0.897); **V3** Go (*p*: 0.978); V4 B (*p*: 0.991); V5 S (*p*: 0.994) **Y axis:** Point with **highest error:** Sn (*p* < 0.004) in V1. En V2, V3, V4, V5 all the point *p* > 0.05. Point with **lowest error**: V1 Lis (*p*: 0.974); V2 Ar (*p*: 0.949); **V3** Pog (*p*: 0.952); V4 Ll (*p*: 0.985); V5 Po (*p*: 1.00)
Lee J, et al. [10]	BCNN-based model CNN, DL	19 points 15 Hard tissue 4 Soft tissue	150 training - validation 250 test	ML carried out by two expert specialists in orthodontics, junior and senior	Mean SDR: 82.11% Cephalometric point with the **highest** success rate: Ll 97.33% Cephalometric point with the **lowest** success rate: A point 52%	Mean MRE: 1.53 mm +/- 1.74 Cephalometric point with the **highest** mean error: Pog’ 2.62 mm +/- 2.07 Cephalometric point with the **lowest** mean error: S 0.86 mm +/- 1.92	-
Yao J, et al. [55]	Algorithm based on a global detection module and a locally modified module CNN, DL	37 points 26 Hard tissue 11 Soft tissue	312 training 100 validation 100 test	ML performed by two expert orthodontic specialists	Mean SDR: 97.30% Cephalometric point with the **highest** success rate: Mes 100% Cephalometric point with the **lowest** success rate: Go and Pt 65%	Mean MRE: 1.04 mm+/- 0.89 Cephalometric point with the **highest** mean error: Pog’ 2.03 mm +/- 5.95 Cephalometric point with the **lowest** mean error: Prn 0.5 mm +/- 0.32	SDR clinical range 1 mm Mean SDR: 54.05% Cephalometric point with the **highest** success rate: Prn 94% Cephalometric point with the **lowest** success rate: Pt 27%
Zhao C, et al. [54]	Multi-Scale YOLOV3 CNN, DL	19 points 15 Hard tissue 4 Soft tissue	1950 training ¿? - validation 150 test	Automatic localization performed by the classic YOLOv3	Mean SDR MS-YOLOv3: 80% Cephalometric point with the **highest** success rate: S and PNS 95.33% Cephalometric point with the **lowest** success rate: Go 55.33% Mean SDR YOLOv3: 76.55% Cephalometric point with the **highest** success rate: S 91.33% Cephalometric point with the **lowest** success rate: Go 52%	Mean MRE MS-YOLOv3: - Cephalometric point with the **highest** mean error: Go 2.43 mm +/- 1.56 Cephalometric point with the **lowest** mean error: Pog’ 1.13 mm +/- 0.62 Mean MRE YOLOv3: - Cephalometric point with the **highest** mean error: Pog’ 3.25 mm +/- 1.79 Cephalometric point with the **lowest** mean error: ANS 1.46 mm +/- 0.97	-
Ristau B, et al. [50]	AudaxCeph CNN, DL	13 points Hard tissue	- No algorithm development	ML performed by 2 orthodontists with 31 and 34 years of experience	-	-	Mean difference Reviewer-AudaxCeph X, Y axis (mean + SD of error) **(regarding *p* > 0.05)** **X axis:** Deviation AI: Po 2.6 mm +/- 2.1. *p* = 0.88. **Y axis:** Deviation AI: L1 apex 2.0 mm +/- 1.3. *p* = 0.46. Or 1.8 +/- 1.2 *p* = 0.11, Po 2.3 mm +/- 2.2 *p* = 0.36 Difference Reviewer- AudaxCeph X, Y axis (%) **X axis**: Po 35.8% **Y axis:** L1 apex 40%
Ramadan R, et al. [49]	CNN-based model creating ROIs (regions of interest) and feature extraction with ResNet50 CNN, DL	19 points 15 Hard tissue 4 Soft tissue	150 training 250 test (150 testset, 100 testset2)	ML performed by two expert orthodontic specialists	Mean SDR Testset 1: 90.39% Cephalometric point with the **highest** success rate: S 98% Cephalometric point with the **lowest** success rate: A point 70.1% Mean SDR Testset 2: 82.66% Cephalometric point with the **highest** success rate: S and upper incisal edge 97.2% Cephalometric point with the **lowest** success rate: Ll 25.4%	Mean MRE Testset 1: 1.23 mm +/- 0.73 Cephalometric point with the **highest** mean error: A point 2.1 mm +/- 1.43 Cephalometric point with the **lowest** mean error: S 0.33 mm +/- 0.12 Mean MRE Testset 2: 1.37 +/- 0.88 mm Cephalometric point with the **highest** mean error: Ll 3.51 mm +/- 2.01 Cephalometric point with the **lowest** mean error: Upper incisor edge 0.32 mm +/- 0.22	
Hwang H, et al. [59]	YOLO version 3 CNN, DL	19 points 15 Hard tissue 4 Soft tissue	1.983 training 200 test	ML performed by 1 examiner with 30 years of experience	Mean SDR: 75.45% Cephalometric point with the **highest** success rate: S 96% Cephalometric point with the **lowest** success rate: Go 38%	Mean MRE: 1.76 mm +/- 2.16 Cephalometric point with the **highest** mean error: Or 2.84 mm +/- 5.91 Cephalometric point with the **lowest** mean error: Ll 1.04 mm +/- 0.60	
Ye H, et al. [48]	MyOrthoX Angelalign Digident CNN, DL	32 points 21 Hard tissue 11 Soft tissue	- No algorithm development	ML performed by an orthodontist, and verified by 2 more orthodontists	Mean SDR Angelalign: 93.09% Cephalometric point with the highest success rate: Sn, Ul, Ll, Si, Pog’, Me’, Gn’, S, Ptm, U1, Pog, Gn, Me 98% Cephalometric point with the lowest success rate: Po and Pt 79% Mean SDR MyOrthoX: 89.99% Cephalometric point with the highest success rate: Gn, Sn, Pog’, Gn’, S, PNS, Pog 100% Cephalometric point with the lowest success rate: UIA 35% Mean SDR Digident: 87.53% Cephalometric point with the highest success rate: Ul, Ll, Pog’, S, PNS, Pog, Gn, Me, Pcd 100% Cephalometric point with the lowest success rate: ANS 35%	Mean MRE Angelalign: 0.80 mm +/- 0.26 Cephalometric point with the **highest** mean error: Pt 1.49 mm +/- 1.24 Cephalometric point with the **lowest** mean error: Go 0.46 mm +/- 0.54 Mean MRE MyOrthoX: 0.97 mm +/- 0.51 Cephalometric point with the **highest** mean error: UIA 2.39 mm +/- 1.05 Cephalometric point with the **lowest** mean error: Sn 0.55 mm +/- 0.32 Mean MRE Digident: 1.11 mm +/- 0.48 Cephalometric point with the **highest** mean error: Prn 2.34 mm +/- 1.57 Cephalometric point with the **lowest** mean error: Gn 0.43 mm +/- 0.29	SDR clinical range <1 mm: Mean SDR Angelalign: 78.08% Cephalometric point with the **highest** success rate: Go 98% Cephalometric point with the **lowest** success rate: Pt 40% Mean SDR MyOrthoX: 67.02% Cephalometric point with the **highest** success rate: Sn 91% Cephalometric point with the **lowest** success rate:UIA 5% Mean SDR Digident: 59.13% Cephalometric point with the **highest** success rate: Pog, Me 95% Cephalometric point with the **lowest** success rate: Prn 19% Mean time Manual group: 153.47 s +/- 14.83 AI group: **Angelalign**: 5.18 s +/- 0.19. **MyOrthoX**: 1.08 s +/- 0.12. **Digident**: 5.60 s +/- 0.20
Uğurlu M [56]	CranioCatch CNN, DL	21 points 17 Hard tissue 4 Soft tissue	1.360 training 140 validation 180 test	ML performed by 1 examiner with 9 years of experience	Mean SDR: 76.2% Cephalometric point with the **highest** success rate: S 98.3% Cephalometric point with the **lowest** success rate: Go 48.3%	Mean MRE: 3.40 mm +/- 1.57 Cephalometric point with the **highest** mean error: Go 8.30 mm +/- 2.98 Cephalometric point with the **lowest** mean error: S 0.62 mm +/- 0.43	
Davidovitch M, et al. [57]	Algoceph CNN, DL	21 points 15 Hard tissue 6 Soft tissue	- No algorithm development	ML performed by 7 orthodontic teachers, 9 3rd year residents and 10 1st year residents, and 4 technicians from the imaging center	Mean SDR: 85.72%	Mean MRE: - Cephalometric point with the **highest** mean error: Pog’ in **Y axis** 2.67 +/- 2.55 mm Cephalometric point with the **lowest** mean error: Soft nose 0.01 mm +/- 0.75 and B point 0.01 +/- 0.65 mm in **Y axis.**	Significant difference in points between AI and MT according to X, Y coordinate axes **X axis**: Or 1.07 mm +/- 1.29, Ptm 0.99 mm +/- 0.98, Ba 1.03 mm +/- 0.90 **Y axis:** Pog’ 2.67 mm +/- 2.55, Ul 1.11 mm +/- 1.16, Po 1.14 mm +/- 1.41

SDR: successful detection rate; MRE: mean radial error; SD: standard deviation; DL: deep learning; ML: manual localization; CNN: convolutional neural networks; ROI: regions of interest; V1: −30% brightness, +30% contrast; V2: −15% brightness, +15% contrast; V3: original; V4: +15% brightness, −15% contrast; V5: +30% brightness, −30% contrast; Ar: articular; A: A point; Ba: basion; B: supramentonal; ANS: anterior nasal spine; PNS: posterior nasal spine; Go: gonion; Gn’: gnation; L1 apex: lower incisor apex; Ul: upper lip; Ll: lower lip; Lis y LIB: lower incisal edge; Me: chain; Me’ and Mes: soft chin; Root Mx6: root 1st maxilar molar; Root Md6: distal first mandibular molar root; Crown Mx1: maxillary incisor crown; Na: nasion; Or: orbitale; Pt: pterygoid; Prn: pronasal; Pog: pogonion; Pog’: soft pogonion; Po: porion,; Ptm: pterygomaxillary fissure; Pcd: posterior condyle; Sn: subnasale; Si: mentolabial groove; S: sella; U1: upper incisor; UIA: upper incisor apex; UIB: upper incisal margin.

## References

[1] Kiełczykowski M., Kamiński K., Perkowski K., Zadurska M., Czochrowska E. (2023). Application of Artificial Intelligence (AI) in a Cephalometric Analysis: A Narrative Review. Diagnostics.

[2] Monill-González A., Rovira-Calatayud L., d’Oliveira N.G., Ustrell-Torrent J.M. (2021). Artificial intelligence in orthodontics: Where are we now? A scoping review. Orthod. Craniofacial Res..

[3] Lee J.G., Jun S., Cho Y.W., Lee H., Kim G.B., Seo J.B., Beom J., Kim N. (2017). Deep Learning in Medical Imaging: General Overview. Korean J. Radiol..

[4] Bichu Y.M., Hansa I., Bichu A.Y., Premjani P., Flores-Mir C., Vaid N.R. (2021). Applications of artificial intelligence and machine learning in orthodontics: A scoping review. Prog. Orthod..

[5] Kaul V., Enslin S., Gross S.A. (2020). History of artificial intelligence in medicine. Gastrointest. Endosc..

[6] Malik P.A., Pathania M., Rathaur V.K. (2019). Overview of artificial intelligence in medicine. J. Fam. Med. Prim. Care.

[7] Wong K.F., Lam X.Y., Jiang Y., Yeung A.W.K., Lin Y. (2023). Artificial intelligence in orthodontics and orthognathic surgery: A bibliometric analysis of the 100 most-cited articles. Head Face Med..

[8] Yamashiro T., Ko C.C. (2021). Artificial intelligence and machine learning in orthodontics. Orthod. Craniofacial Res..

[9] Le V.N.T., Kang J., Oh I.S., Kim J.G., Yang Y.M., Lee D.W. (2022). Effectiveness of Human-Artificial Intelligence Collaboration in Cephalometric Landmark Detection. J. Pers. Med..

[10] Lee J.H., Yu H.J., Kim M.J., Kim J.W., Choi J. (2020). Automated cephalometric landmark detection with confidence regions using Bayesian convolutional neural networks. BMC Oral Health.

[11] Kim H., Shim E., Park J., Kim Y.J., Lee U., Kim Y. (2020). Web-based fully automated cephalometric analysis by deep learning. Comput. Methods Programs Biomed..

[12] Leonardi R., Giordano D., Maiorana F., Spampinato C. (2008). Automatic Cephalometric Analysis a Systematic Review. Angle Orthod..

[13] Mehta S., Suhail Y., Nelson J., Upadhyay M. (2021). Artificial Intelligence for radiographic image analysis. Semin. Orthod..

[14] Liu J., Zhang C., Shan Z., Liu J., Zhang C., Shan Z. (2023). Application of Artificial Intelligence in Orthodontics, Current State and Future Perspectives. Healthcare.

[15] Moher D., Liberati A., Tetzlaff J., Altman D.G. (2010). Preferred reporting items for systematic reviews and meta-analyses, the PRISMA statement. Int. J. Surg..

[16] Whiting P.F., Rutjes A.W., Westwood M.E., Mallett S., Deeks J.J., Reitsma J.B., Leeflang M.M., Sterne J.A., Bossuyt P.M. (2011). QUADAS-2 Group. QUADAS-2: A revised tool for the quality assessment of diagnostic accuracy studies. Ann. Intern. Med..

[17] Wells G.A., Shea B., O’Connell D., Peterson J., Welch V., Losos M., Tugwell P. (2014). The Newcastle-Ottawa Scale (NOS) for Assessing the Quality of Nonrandomized Studies in Meta-Analyses.

[18] Wang C.W., Huang C.T., Hsieh M.C., Li C.H., Chang S.W., Li W.C., Remy V., Raphael M., Sebastien J., Pierre G. (2015). Evaluation and Comparison of Anatomical Landmark Detection Methods for Cephalometric X-Ray Images: A Grand Challenge. IEEE Trans. Med. Imaging.

[19] Hwang H.W., Park J.H., Moon J.H., Yu Y., Kim H., Her S.B., Srinivasan G., Aljanabi M.N., Donatelli R.E., Lee S.J. (2020). Automated identification of cephalometric landmarks, Part 2-Might it be better than human?. Angle Orthod..

[20] Gong B.W., Chang S., Zuo F.F., Xie X.J., Wang S.F., Wang Y.J., Sun Y.Y., Guan X.C., Bai Y.X. (2023). Automated cephalometric landmark identification and location based on convolutional neural network. Zhonghua Kou Qiang Yi Xue Za Zhi.

[21] Moreno M., Gebeile-Chauty S. (2022). Comparative study of two software for the detection of cephalometric landmarks by artificial intelligence. L’Orthodontie Française.

[22] Chen J., Che H., Sun J., Rao Y., Wu J. An automatic cephalometric landmark detection method based on heatmap regression and Monte Carlo dropout. Proceedings of the 2023 45th Annual International Conference of the IEEE Engineering in Medicine & Biology Society (EMBC).

[23] King C.H., Wang Y.L., Lin W.Y., Tsai C.L. Automatic Cephalometric Landmark Detection on X-Ray Images Using Object Detection. Proceedings of the 2022 IEEE 19th International Symposium on Biomedical Imaging (ISBI).

[24] Du D., Ren T., Chen C., Jiang Y., Song G., Li Q., Niu J. Anatomical Landmarks Annotation on 2D Lateral Cephalograms with Channel Attention. Proceedings of the 2022 22nd IEEE International Symposium on Cluster, Cloud and Internet Computing (CCGrid).

[25] Fajar A., Pangestu G., Sarno R., Ardani I.G.A.W. Cephalometric Landmark Detection on Cephalograms using Regression CNN. Proceedings of the 2022 5th International Conference on Information and Communications Technology (ICOIACT).

[26] Rashmi S., Srinath S., Rakshitha R., Poornima B.V. Extended Template Matching method for Region of Interest Extraction in Cephalometric Landmarks Annotation. Proceedings of the 2022 IEEE 9th Uttar Pradesh Section International Conference on Electrical, Electronics and Computer Engineering (UPCON).

[27] Song Y., Qiao X., Iwamoto Y., Chen Y.W. A Teacher-Student Learning Based On Composed Ground-Truth Images For Accurate Cephalometric Landmark Detection. Proceedings of the 2021 IEEE International Conference on Image Processing (ICIP).

[28] Reddy P.K., Kanakatte A., Gubbi J., Poduval M., Ghose A., Purushothaman B. Anatomical Landmark Detection using Deep Appearance-Context Network. Proceedings of the 2021 43rd Annual International Conference of the IEEE Engineering in Medicine & Biology Society (EMBC).

[29] Zhang Q., Guo J., He T., Yao J., Tang W., Yi Z. A Novel Landmark Detection Method for Cephalometric Measurement. Proceedings of the 2021 IEEE International Conference on Medical Imaging Physics and Engineering (ICMIPE).

[30] Goutham E.N.D., Vasamsetti S., Kishore P.V.V., Sardana H.K. Automatic Localization of Landmarks in Cephalometric Images Via Modified U-Net. Proceedings of the 2019 10th International Conference on Computing, Communication and Networking Technologies (ICCCNT).

[31] Tabata L.C., Nyirenda C.N., Faster R.-C.N.N. Based Cephalometric Landmarks Detection. Proceedings of the 2021 IEEE AFRICON.

[32] El-Dawlatly M., Attia K.H., Abdelghaffar A.Y., Mostafa Y.A., Abd El-Ghafour M. (2024). Preciseness of artificial intelligence for lateral cephalometric measurements. J. Orofac. Orthop..

[33] Lee J., Bae S.R., Noh H.K. (2023). Commercial artificial intelligence lateral cephalometric analysis: Part 1—The possibility of replacing manual landmarking with artificial intelligence service. J. Clin. Pediatr. Dent..

[34] Lee J., Bae S.R., Noh H.K. (2023). Commercial artificial intelligence lateral cephalometric analysis: Part 2—Effects of human examiners on artificial intelligence performance, a pilot study. J. Clin. Pediatr. Dent..

[35] Jeon S., Lee K.C. (2021). Comparison of cephalometric measurements between conventional and automatic cephalometric analysis using convolutional neural network. Prog. Orthod..

[36] Vithanaarachchi N., Chandrasiri A., Nawarathna L. (2020). A comparison of cephalometric measurements obtained using conventional and digital methods. Ceylon Med. J..

[37] Kang S., Kim I., Kim Y.J., Kim N., Baek S.H., Sung S.J. (2024). Accuracy and clinical validity of automated cephalometric analysis using convolutional neural networks. Orthod. Craniofacial Res..

[38] Kumar M., Kumari S., Chandna A., Singh A., Kumar K., Punita H. (2020). Comparative Evaluation of CephNinja for Android and NemoCeph for Computer for Cephalometric Analysis: A Study to Evaluate the Diagnostic Performance of CephNinja for Cephalometric Analysis. J. Int. Soc. Prev. Community Dent..

[39] Nishimoto S., Sotsuka Y., Kawai K., Ishise H., Kakibuchi M. (2019). Personal computer-based cephalometric landmark detection with deep learning, using cephalograms on the internet. J. Craniofacial Surg..

[40] Gomez-Trenado G., Mesejo P., Cordon O. (2023). Cascade of convolutional models for few-shot automatic cephalometric landmarks localization. Eng. Appl. Artif. Intell..

[41] Oh K., Oh I.S., Le V.N.T., Lee D.W. (2021). Deep Anatomical Context Feature Learning for Cephalometric Landmark Detection. IEEE J. Biomed. Health Inform..

[42] Park J.H., Hwang H.W., Moon J.H., Yu Y., Kim H., Her S.B., Girish S., Noori A.A.M., Richard E.D., Lee S.-J. (2019). Automated identification of cephalometric landmarks: Part 1—Comparisons between the latest deep-learning methods YOLOV3 and SSD. Angle Orthod..

[43] Jiang F., Guo Y., Yang C., Zhou Y., Lin Y., Cheng F., Shuqi Q., Qingchen F., Li J. (2023). Artificial intelligence system for automated landmark localization and analysis of cephalometry. Dentomaxillofacial Radiol..

[44] Wang X., Rigall E., Chen Q., Zhang S., Dong J. (2022). Efficient and Stable Cephalometric Landmark Localization Using Two-Stage Heatmaps’ Regression. IEEE Trans. Instrum. Meas..

[45] Lu G., Zhang Y., Kong Y., Zhang C., Coatrieux J.L., Shu H. (2022). Landmark Localization for Cephalometric Analysis Using Multiscale Image Patch-Based Graph Convolutional Networks. IEEE J. Biomed. Health Inform..

[46] Qian J., Luo W., Cheng M., Tao Y., Lin J., Lin H. (2020). CephaNN: A Multi-Head Attention Network for Cephalometric Landmark Detection. IEEE Access.

[47] Neeraja R., Anbarasi L.J. (2023). CephXNet: A Deep Convolutional Squeeze-and-Excitation Model for Landmark Prediction on Lateral Cephalograms. IEEE Access.

[48] Ye H., Cheng Z., Ungvijanpunya N., Chen W., Cao L., Gou Y. (2023). Is automatic cephalometric software using artificial intelligence better than orthodontist experts in landmark identification?. BMC Oral Health.

[49] Ramadan R.A., Khedr A.Y., Yadav K., Alreshidi E.J., Sharif M.H., Azar A.T., Kamberaj H. (2022). Convolution neural network based automatic localization of landmarks on lateral x-ray images. Multimed. Tools Appl..

[50] Ristau B., Coreil M., Chapple A., Armbruster P., Ballard R. (2022). Comparison of AudaxCeph^®^’s fully automated cephalometric tracing technology to a semi-automated approach by human examiners. Int. Orthod..

[51] Kim J., Kim I., Kim Y.J., Kim M., Cho J.H., Hong M., Kyung-Hwa K., Sung-Hoon L., Su-Jung K., Ho K.Y. (2021). Accuracy of automated identification of lateral cephalometric landmarks using cascade convolutional neural networks on lateral cephalograms from nationwide multi-centres. Orthod. Craniofacial Res..

[52] Bulatova G., Kusnoto B., Grace V., Tsay T.P., Avenetti D.M., Sanchez F.J.C. (2021). Assessment of automatic cephalometric landmark identification using artificial intelligence. Orthod. Craniofacial Res..

[53] Santos Menezes L.D., Silva T.P., Lima dos Santos M.A., Hughes M.M., Reis Mariano Souza S.D., Leite Ribeiro P.M., Luiz F.P.H., Takeshita W.M. (2023). Assessment of landmark detection in cephalometric radiographs with different conditions of brightness and contrast using the an artificial intelligence software. Dentomaxillofacial Radiol..

[54] Zhao C.Y., Yuan Z.B., Luo S.C., Wang W.J., Ren Z., Yao X.F., Wu T. (2023). Automatic recognition of cephalometric landmarks via multi-scale sampling strategy. Heliyon.

[55] Yao J., Zeng W., He T., Zhou S., Zhang Y., Guo J., Tang W. (2022). Automatic localization of cephalometric landmarks based on convolutional neural network. Am. J. Orthod. Dentofac. Orthop..

[56] Uğurlu M. (2022). Performance of a Convolutional Neural Network—Based Artificial Intelligence Algorithm for Automatic Cephalometric Landmark Detection. Turk. J. Orthod..

[57] Davidovitch M., Sella-Tunis T., Abramovicz L., Reiter S., Matalon S., Shpack N. (2022). Verification of convolutional Neural Network Cephalometric Landmark Identification. Appl. Sci..

[58] Kim Y.H., Lee C., Ha E.G., Choi Y.J., Han S.S. (2021). A fully deep learning model for the automatic identification of cephalometric landmarks. Imaging Sci. Dent..

[59] Hwang H.W.W., Moon J.H.H., Kim M.G.G., Donatelli R.E.E., Lee S.J.J. (2021). Evaluation of automated cephalometric analysis based on the latest deep learning method. Angle Orthod..

[60] Hwang J.J., Jung Y.H., Cho B.H., Heo M.S. (2019). An overview of deep learning in the field of dentistry. Imaging Sci. Dent..

[61] Durão A.P.R., Morosolli A., Pittayapat P., Bolstad N., Ferreira A.P., Jacobs R. (2015). Cephalometric landmark variability among orthodontists and dentomaxillofacial radiologists: A comparative study. Imaging Sci. Dent..

[62] Miloro M., Borba A.M., Ribeiro-Junior O., Naclério-Homem M.G., Jungner M. (2014). Is there consistency in cephalometric landmark identification amongst oral and maxillofacial surgeons?. Int. J. Oral Maxillofac. Surg..

